# Continuous chest compressions with a simultaneous triggered ventilator in the Munich Emergency Medical Services: a case series

**DOI:** 10.3205/000272

**Published:** 2019-06-26

**Authors:** Stefan J. Schaller, Sonja Altmann, Annalise Unsworth, Gerhard Schneider, Viktoria Bogner-Flatz, Thomas Paul, Petra Hoppmann, Karl-Georg Kanz

**Affiliations:** 1 Department of Anesthesiology and Intensive Care, School of Medicine, Klinikum rechts der Isar, Technical University of Munich, Germany; 2 Faculty of Medicine, University of New South Wales, Kensington, NSW, Australia; 3 Department of Trauma Surgery, Ludwig-Maximilians-University Munich, Germany; 4 Board of Directors, Emergency Medical Services, Munich, Germany; 5 Emergency Medical Services, Munich Fire Department, Munich, Germany; 6 Department of Cardiology, School of Medicine, Klinikum rechts der Isar, Technical University of Munich, Germany; 7 Department of Trauma Surgery, School of Medicine, Klinikum rechts der Isar, Technical University of Munich, Germany

**Keywords:** cardiopulmonary resuscitation, emergency therapy, ventilation, ventilators, emergency medical services

## Abstract

**Background:** Mechanical chest compression devices are commonly used providing a constant force and frequency of chest compression during cardiopulmonary resuscitation. However, there are currently no recommendations on ventilation during cardiopulmonary resuscitation with a mechanical chest compression device using continuous mode. An effective method for ventilation in such scenarios might be a triggered oxygen-powered resuscitator.

**Methods:** We report seven cardiopulmonary resuscitation cases from the Munich Emergency Medical Service where mechanical chest compression devices in continuous mode were used with an oxygen-powered resuscitator. In each case, the resuscitator (Oxylator^®^) was running in automatic mode delivering a breath during the decompression phase of the chest compressions at a frequency of 100 per minute. End-tidal carbon dioxide and pulse oximetry were measured. Additional data was collected from the resuscitation protocol of each patient.

**Results:** End-tidal carbon dioxide was available in all cases while oxygen saturation only in four. Five patients had a return of spontaneous circulation. Based on the end-tidal carbon dioxide values of each of the cases, the resuscitator did not seem to cause hyperventilation and suggests that good-quality cardiopulmonary resuscitation was delivered.

**Conclusions:** Continuous chest compressions using a mechanical chest compression device and simultaneous synchronized ventilation using an oxygen-powered resuscitator in an automatic triggering mode might be feasible during cardiopulmonary resuscitation.

## Zusammenfassung

**Hintergrund:** Geräte zur mechanischen Thoraxkompression werden heute routinemäßig eingesetzt, unter anderem, weil sie eine kontinuierliche Kompressionsstärke, -tiefe und -frequenz während einer kardiopulmonalen Reanimation ermöglichen. Bezüglich der Beatmung bei Reanimation mittels Thoraxkompressionsgerät in kontinuierlichem Modus gibt es aktuell keine Empfehlungen. Dafür wäre ein mit Sauerstoff betriebener triggerbarer Ventilator eventuell geeignet.

**Methode:** Wir berichten von sieben Reanimationen im Münchner Rettungsdienst, die mittels Thoraxkompressionsgerät im kontinuierlichen Modus durchgeführt wurden und bei denen gleichzeitig ein mit Sauerstoff betriebener, automatisch auslösender Notfallventilator zur Anwendung kam. In allen sieben Fällen handelte es sich dabei um den Oxylator^®^, der im automatischen Modus jedes Mal in der Dekompressionsphase der Thoraxkompression einen Beatmungshub auslöst. Somit beatmet der Ventilator synchron mit dem Thoraxkompressionsgerät mit einer Beatmungsfrequenz von 100 pro Minute. Als Monitoring dienten endtidales Kohlendioxid und die Sauerstoffsättigung. Weitere Daten wurden den Rettungsdienstprotokollen entnommen.

**Ergebnisse:** Endtidales Kohlendioxid war in allen sieben Fällen messbar, die Sauerstoffsättigung nur in vier. Bei fünf der Patienten konnte eine Wiederherstellung des Kreislaufes erreicht werden. Basierend auf den endtidalen Kohlendioxidwerten kann eine gute Qualität der kardiopulmonalen Reanimation angenommen werden sowie eine Hyperventilation als unwahrscheinlich erachtet werden.

**Fazit:** Während einer kardiopulmonalen Reanimation mittels Thoraxkompressionsgerät im kontinuierlichen Modus war eine Ventilation mit einem sauerstoffbetriebenen, automatisch auslösenden Notfallventilator in sieben Fällen zuverlässig möglich.

## Background

Out-of-hospital cardiac arrest is a common cause of Emergency Medical Service (EMS) notification in Germany. Survival requires immediate cardiopulmonary resuscitation (CPR) [1] with survival rates ranging from 0.3% to 31% [2]. In Munich, the thirty-day survival rate for out-of-hospital cardiac arrest is currently 12.1%. The aim of CPR is to ensure sufficient cerebral and cardiac blood flow applying heart massage and oxygen ventilation with the goal to achieve a return of spontaneous circulation (ROSC). Guidelines for CPR by the European Resuscitation Council recommend a ratio of 30 chest compressions to 2 breaths, at a compression depth of 5 cm and a frequency of 100 compressions per minute [3].

However, even amongst experienced healthcare professionals, chest compressions and ventilation are often insufficient focusing on quality of CPR more recently [4], [5], [6]. Mechanical chest compression devices (MCCDs) allow a constant force and frequency of chest compressions. These devices increase cardiac output and hence cerebral and cardiac perfusion [7], [8]. Maintaining continuous chest compressions is important since small interruptions may have a negative impact on survival and neurological outcomes [9], [10], [11], [12]. Consequently, a ventilation method that does not cause any interruptions in chest compressions or can be used during continuous mode of MCCDs might have positive effects on outcomes.

The Munich EMS has a three-tiered response system for an unconscious person. The three responses are dispatched simultaneously. The first response is a fire engine, equipped with a mechanical chest compression device (LUCAS 2^®^, Physio Control, Lund, Sweden), an automated external defibrillator, and a patient-responsive automatically triggering oxygen-powered resuscitator (Oxylator^®^ HD, CPR Medical Devices Inc., Ontario, Canada) as emergency ventilator. For quality management reasons, a Tidalwave^®^ device with continuous peripheral oxygen saturation (SpO_2_) and end-tidal CO_2_ (etCO_2_) monitoring (Tidalwave^®^, Novametrix Inc. (Phillips) USA/NL (Physio Control, Washington, USA) has been used. This tier is followed by a paramedic ambulance service and a physician-staffed ambulance.

Since there are no recommendations on ventilation during continuous mode of MCCDs [13], [14], we reviewed our data files for CPR cases where an MCCD in continuous mode was used, together with an oxygen-powered resuscitator in automatic triggering mode.

## Methods

This case series presents seven CPR cases of the Munich Fire Department with both a continuous MCCD (LUCAS 2^®^, Physio Control, Washington, USA) and a patient-responsive automatically triggering resuscitator (Oxylator^®^ HD, CPR Medical Devices Inc., Ontario, Canada). In Munich, the basic setting of the MCCD delivers compressions and breaths in a ratio of 30:2. However, in the presented cases the MCCD was – either by mistake or by the physician’s decision – set to automatic mode, thereby delivering continuous chest compressions with a frequency of 100/minute without any interruption.

The Oxylator^®^ HD resuscitation and inhalation management system is a patient-responsive emergency ventilation device that can be used in either automatic or manual mode. In automatic mode, it administers oxygen or air to the patient at a constant flow rate of 30 l/m during the inspiration phase until the airway pressure set is reached, thereupon automatically switching to the passive exhalation phase that lasts until the device registers lack of flow coming from the patient. At that point, the device automatically switches back to the inspiration phase, repeating the cycle. The pressure selection ranges from 15 to 30 cm H_2_O. The Oxylator^®^ technology operates on a “closed loop” system. In case of an MCCD applied, the Oxylator^®^ HD will trigger the inspiration phase with every decompression phase.

Data was used retrospectively from the Munich Fire Department Quality Management records. Data included a resuscitation protocol case sheet containing patient and resuscitation characteristics, the rate of chest compressions, the rate of ventilation, peripheral oxygen saturations, and main-stream etCO_2_. This analysis is covered by the ethical approval 508/16 of the ethic committee of the School of Medicine, Technical University of Munich, Munich, Germany.

## Case descriptions

### Case 1

A 65-year-old male patient was found collapsed. The case was initially managed by a paramedic ambulance team. Manual CPR was performed for nine minutes, and a laryngeal mask was inserted. Initial rhythm analysis showed ventricular fibrillation, and the patient was defibrillated three times. Upon arrival of the fire service, an MCCD and a resuscitator were attached. SpO_2_ and etCO_2_ were measured and recorded.

**Figure 1:**
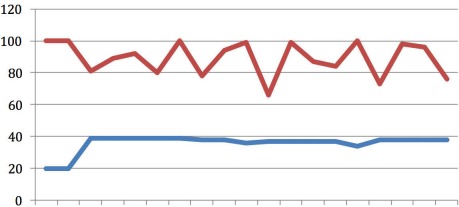
Case 1 X-axis representing time in 16 sec intervals; upper line (red): CO_2_ (mmHg); lower line (blue): Resp (br/m)

Figure 1 (Fig. 1) illustrates the first three minutes of resuscitation by the fire service prior to the attachment of the MCCD. In this initial period, ventilation was matched to manual chest compression. The respiration frequency varied with the manual CPR, ranging between 75 and 100 breaths per minute. EtCO_2_ was 20 mmHg for the first 30 seconds and then increased to 40 mmHg for the remainder of the recording. After 25 minutes of CPR, return of spontaneous circulation (ROSC) occurred and the patient was transported to a nearby hospital.

### Case 2

A 64-year-old unconscious male patient with cardiac arrest. The time between EMS notification and arrival of the fire service was eight minutes. An MCCD and a facemask together with a resuscitator were attached, and CPR was commenced.

**Figure 2:**
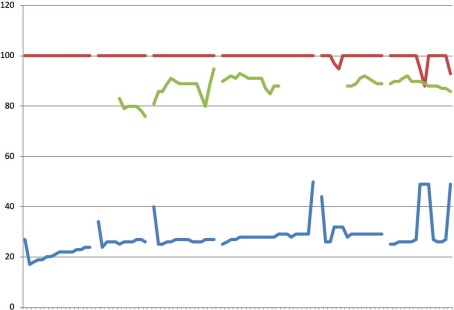
Case 2 X-axis representing time in 16 sec intervals; upper line (red): CO_2_ (mmHg); middle line (green): Resp (br/m); lower line (blue): SpO_2_ (%)

Figure 2 (Fig. 2) illustrates fifteen minutes of continuous CPR. Interruptions in the recording reflect interruptions in CPR during heart rhythm analysis and endotracheal intubation. The oxygen saturation ranged between 80–97%; however recordings included accidental removal of the pulse oximeter finger clip, poor circulatory status, hypothermia and vasoconstrictive medications. The etCO_2_ remained between 20 and 30 mmHg, which suggests sufficient CPR. EtCO_2_ increased after each interruption in CPR, as the CO_2_ accumulates due to decreased exhalation and lack of blood circulation. Defibrillation was not indicated, and upon arrival of the physician-staffed ambulance service, the patient was intubated and epinephrine administered. After 27 minutes, CPR was discontinued and the patient was declared deceased.

### Case 3

A 79-year-old male patient collapsed outside. A paramedic ambulance was first on scene, and manual CPR was commenced and continued for 11 minutes. After the arrival of the fire service, the LUCAS 2^®^ and Oxylator^®^ HD were attached.

**Figure 3:**
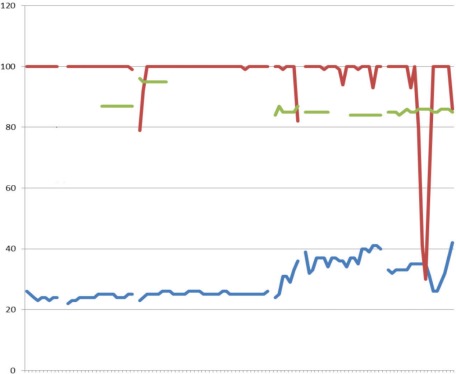
Case 3 X-axis representing time in 16 sec intervals; upper line (red): CO_2_ (mmHg); middle line (green): Resp (br/m); lower line (blue): SpO_2_ (%)

In Figure 3 (Fig. 3), the ventilator curve demonstrates ventilation with a frequency of 100 breaths per minute, which is identical to the compression rate. The periodic decrease in respiratory rate was mostly due to an airway leak. Oxygen saturation was recorded intermittently, however remained between 83% and 95%. The etCO_2_ was between 22–42 mmHg, which is consistent with good CPR. The increase at the end of the recording could be an early indicator of an ROSC. The patient was intubated by the physician and required defibrillation and intravenous epinephrine. After 40 minutes the patient had ROSC and was transported to a nearby hospital.

### Case 4

**Figure 4:**
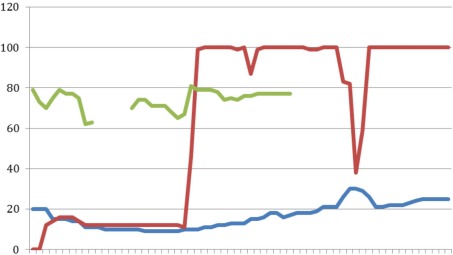
Case 4 X-axis representing time in 16 sec intervals; upper line (red): CO_2_ (mmHg); middle line (green): Resp (br/m); lower line (blue): SpO_2_ (%)

A 55-year-old intoxicated male patient collapsed outside. The paramedic ambulance was first on scene, and initial resuscitation was commenced. Initially, the patient was ventilated manually via a facemask and bag. Upon arrival of the fire service, LUCAS 2^®^ and Oxylator^®^ HD were attached (Figure 4 (Fig. 4)).

In this case the patient was ventilated manually for the first three minutes with a respiratory frequency of 16–19 breaths per minute. Initially, etCO_2_ was 20 mmHg decreasing to 15 mmHg in the first three minutes. Oxygen saturation was between 60% and 80%. After three minutes the respiratory rate increased to 100 breaths per minute by activating the automatic mode of the Oxylator^®^ HD. Later on, the respiratory rate decreased to 30 breaths/minute, due to a leak or airway obstruction. The end-tidal CO_2_ increased when in automatic mode to 15–30 mmHg. The oxygen saturation was recorded for a short period of time and was 79%. The patient was defibrillated twice, and epinephrine was administered. After 30 minutes of CPR, the patient was declared deceased by the emergency physician.

### Case 5

A 45-year-old unconscious male patient with cardiac arrest. The initial response and resuscitation was conducted by the ambulance service. The fire service arrived and attached an MCCD and resuscitator to the inserted laryngeal mask.

**Figure 5:**
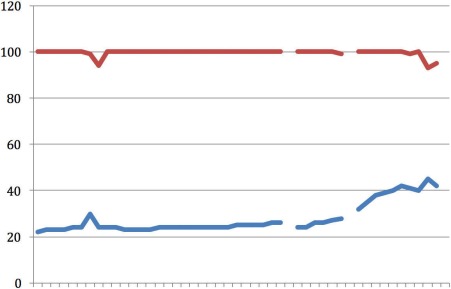
Figure 5. Case 5 X-axis representing time in 16 sec intervals; upper line (red): CO_2_ (mmHg); lower line (blue): Resp (br/m)

Figure 5 (Fig. 5) illustrates the period of continuous resuscitation between 18 and 25 minutes. The ventilation frequency was constant at 100 breaths per minute. Between 18 and 23 minutes, etCO_2_ was 21–30 mmHg, indicating good-quality CPR. After 23 minutes etCO_2_ increased to 43 mmHg, which might be an early indicator of ROSC. Pulse oximetry was not performed or was unable to adequately measure saturation. Initial rhythm strip analysis demonstrated ventricular fibrillation. The patient was defibrillated eight times and received multiple doses of intravenous epinephrine. After 25 minutes, the patient had ROSC and was transported to a nearby hospital.

### Case 6

**Figure 6:**
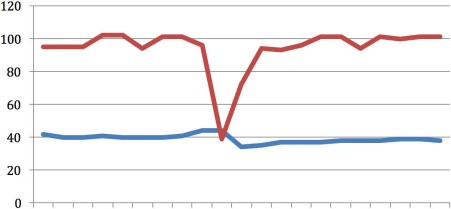
Case 6 X-axis representing time in 16 sec intervals; upper line (red): CO_2_ (mmHg); lower line (blue): Resp (br/m)

A 65-year-old male patient had a witnessed collapse and severe chest pain. The ambulance service initiated manual CPR. Initial rhythm strip analysis demonstrated asystole. After eight minutes, the fire service arrived at the site. Following 20 minutes of manual CPR, an MCCD and a resuscitator were attached (Figure 6 (Fig. 6)).

Ventilation frequency varied around 100 breaths per minute, despite being attached to an MCCD and resuscitator. For approximately 36 seconds there was a decrease in the respiratory rate to 40 breaths per minute. This may be due to a leak in airway management, airway obstruction or the patient requiring a higher airway pressure than 15 cm H_2_O. Smaller variances in the respiratory rate (down to approx. 95 br/min for some seconds) occurred several times. Nevertheless, the etCO_2_ was almost constant around 40 mmHg. Epinephrine was administered several times. After 24 minutes of CPR, the patient had ROSC and was transported to a nearby hospital. A pulmonary embolism was diagnosed, and despite thrombolysis, the patient was subsequently declared deceased.

### Case 7

**Figure 7:**
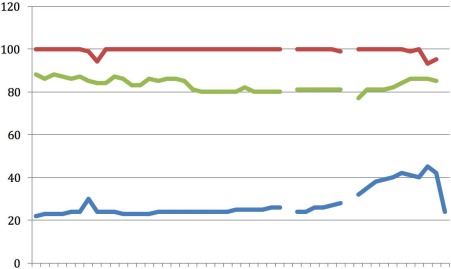
Case 7 X-axis representing time in 16 sec intervals; upper line (red): CO_2_ (mmHg); middle line (green): Resp (br/m); lower line (blue): SpO_2_ (%)

An 84-year-old male patient collapsed outside. A passer-by witnessed the incident, notified emergency services and commenced CPR. The time between call and arrival of the fire service was eight minutes. Initial rhythm strip analysis demonstrated asystole. The resuscitator was attached immediately to a facemask by a firefighter (Figure 7 (Fig. 7)).

The patient received a ventilation rate of 100 breaths per minute. This rate corresponds with the MCCD frequency, and ventilation was being triggered in each decompression phase. The drop in the ventilation rate in the initial part was due to a leak in the airway circuit. During the period of automatic ventilation, oxygen saturation was between 80 and 90%. Thus, the patient was adequately ventilated. The etCO_2_ ranged between 22–30 mmHg from the beginning of the recording till 23:28 minutes after, suggesting good-quality CPR with adequate circulation. During resuscitation, the patient received intravenous epinephrine. After 23:28 min there was an increase in etCO_2_ up to 43 mmHg, an early indicator of ROSC. The steep decrease afterwards was due to the cessation of ventilation and chest compressions, and hence a cessation in CO_2_ exhalation. The patient had an ROSC after 25 minutes and was transported to a nearby hospital.

## Discussion

During CPR, interruptions of chest compressions or lung hyperventilation are common [15]. A useful alternative to manual CPR might be the combination of MCCD in continuous mode with passive ventilation [15]. Previous studies have examined the hypothesis that automatic ventilation with high frequency and low airway pressure may benefit CPR outcomes [16], [17], [18], [19]. Klain et al. [16] described high-frequency jet ventilation during CPR, administering 100–500 breaths per minute through percutaneous cannulation of the trachea. Additionally, Bertrand et al. [19] demonstrated that a constant oxygen supply using a Boussignac tube which has an open main lumen and separate microchannels in the tube wall supplying continuous oxygen leads to better peripheral oxygen saturation during continuous CPR. Using this device, continuous chest compressions produced active exhalation through the main lumen and automatic passive inspiration during decompression. However, the method never became popular in the field.

In the current European Resuscitation Council Guidelines there is little emphasis on early tracheal intubation, as it may result in a significant break in chest compressions [3]. However, if the patient is successfully intubated, continuous chest compressions are recommended with simultaneous ventilation. In order to avoid hyperventilation, the respiratory rate should be 10 or fewer breaths per minute, and the tidal volume 6–8 ml/kg ideal body weight [20], [21], [22]. Due to the risk of high inspiratory pressure, it is not clear whether this approach with MCCDs is advisable.

Hyperventilation and a high inspiratory pressure increase the risk of lung barotrauma and simultaneous gastric hyperventilation, which decrease patient survival [20], [23]. Stomach hyperinflation results in regurgitation and aspiration. In animal studies, it has been shown to cause an abdominal compartment syndrome, which reduces pulmonary function and causes hemodynamic instability [23], [24]. Stomach hyperinflation results from a combination of high ventilation pressure, tidal volume, and inspiratory flow rate. Emergency ventilators such as the Oxylator^®^ HD have a lower risk of gastric hyperinflation compared to manual ventilation, as these ventilators have a constant low flow rate and a pressure limit [25]. The argument that continuous ventilation might lead to dead-space ventilation only cannot be confirmed in our patients due to the intermittent measurement of peripheral oxygen saturation. The risk of hyperventilation from continuous breaths was not apparent based on the measured etCO_2_ values either. The etCO_2_ suggests good-quality CPR, and that the patients may have benefited from continuous chest compressions with simultaneous ventilation.

Our results are in accordance with animal studies by Hu et al. [26], who demonstrated that simultaneous automatic ventilation and chest compressions are possible in CPR. In porcine models, CPR with both an Oxylator^®^ at a pressure of 20 cm H_2_O and a flow of 30 l/min, and an Oxylator^®^ at a pressure of 15 cm H_2_O and flow of 20 l/min, had a higher etCO_2_ than manual ventilation. Furthermore, high-frequency mechanical ventilation was more effective than manual ventilation, as it prevented hyperventilation. Additionally, compared to manual ventilation, the Oxylator^®^ with higher pressure and flow resulted in a more effective resuscitation with a higher arterial pO_2_ and a reduced alveolar-arterial gradient [26]. Human studies do not exist so far, however, a pilot study is on its way (NCT03347175). Only a few other animal studies address this issue, two of them using chest compression synchronized ventilation (CCSV) [27], [28], [29]. Whether CCSV is safe (since the maximum inspiratory pressure was set to 60 mbar) and works efficiently in humans or even has advantages cannot be answered yet.

Consequently, we agree with Bernhard et al.: “There is insufficient or missing evidence for the effectiveness of any ventilation strategy and the use of automated mechanical chest compression devices. To the best of our knowledge, there are no clinical studies that focus on effective oxygenation and elimination of carbon dioxide in patients suffering from cardiac arrest who are being treated with automated mechanical chest compression devices” [14].

### Limitations

All cases presented were male patients, and we do not provide any information on patient characteristics such as height and weight which may affect ventilation or chest compressions. Only non-invasive routine parameters were collected, as the data was part of the Munich Fire Department Quality Management records. The Tidal Wave^®^ device measured etCO_2_ and SpO_2_ in eight-second intervals rather than continuously. Additionally, peripheral oxygen saturation was very susceptible to interference due to the finger clip falling off or not being applied by the fire fighters. We can only speculate why a drop or variation in respiratory rate occurred in some scenarios. However, besides patient factors (e.g. emphysema), a lot of interferences are typical in the preclinical setting, such as standing on the breathing hose, folding of the endotracheal tube, or disruption of the MCCD during transportation.

## Conclusion

This case series along with previous animal studies suggests that continuous chest compression using an MCCD and simultaneous, synchronous emergency ventilation is feasible during CPR and should be investigated on a larger scale.

## Abbreviations

- CCSV: Chest compression synchronized ventilation
- CPR: Cardiopulmonary resuscitation
- EMS: Emergency Medical Service
- etCO_2_: End-tidal CO_2_
- MCCD: Mechanical chest compression device
- ROSC: Return of spontaneous circulation
- SpO_2_: Peripheral oxygen saturation

## Notes

### Ethical declaration

This analysis is covered by the ethical approval 508/16 of the ethic committee of the School of Medicine, Technical University of Munich, Munich, Germany. Consent to participation was waived for the retrospective anonymized analysis.

### Authors’ contributions

- SJS and SA contributed equally to this manuscript.
- SJS participated in drafting the manuscript, data interpretation, and revising the report for intellectual content.
- AU participated in drafting the manuscript, data analysis and interpretation, and revising the report for intellectual content.
- SA participated in the idea, planning, data analysis and interpretation, as well as revising the report for intellectual content.
- GS revised the report for intellectual content.
- VBF revised the report for intellectual content.
- TP participated in the idea, data analysis and interpretation.
- KGK participated in the idea, planning, data analysis and interpretation, as well as writing the report.
- All authors read and approved the final manuscript.

### Competing interests

The authors declare that they have no competing interests.
